# Supplementary material for: Association between oral microbiome and seven types of cancers in East Asian population: a two-sample Mendelian randomization analysis
Source: Front Mol Biosci. 2023 Nov 21;10:1327893. doi: 10.3389/fmolb.2023.1327893 (PMC10702768; doi:10.3389/fmolb.2023.1327893)
Supplement: Supplementary file 1 [file DataSheet1.ZIP › Supplementary Materials/MR plots of saliva/saliva═╝/Lung Cancer/pheno.2566_to_lung cancer_forest.pdf]

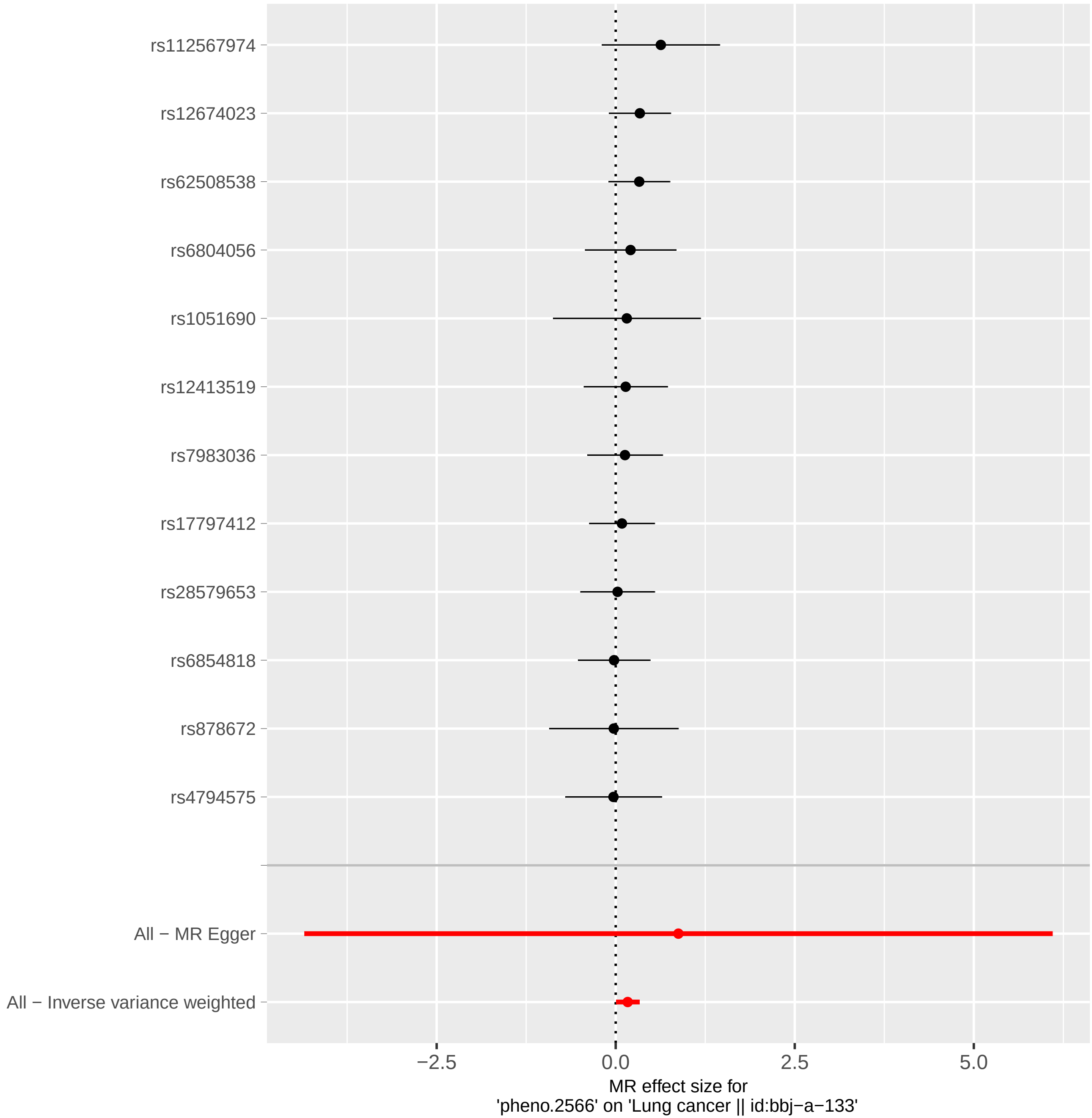
rs112567974
rs12674023
rs62508538
rs6804056
rs1051690
rs12413519
rs7983036
rs17797412
rs28579653
rs6854818
rs878672
rs4794575
All − MR Egger
All − Inverse variance weighted
−2.5
0.0
2.5
5.0
MR effect size for
'pheno.2566' on 'Lung cancer || id:bbj−a−133'
